# Performance evaluation of the QIAstat-Dx gastrointestinal panel 2 for the detection of *Clostridioides difficile* against multiple commercial assays

**DOI:** 10.1128/jcm.00576-25

**Published:** 2025-10-21

**Authors:** Lavannya Sabharwal, Amorina Purpora, Derek Gerstbrein, Kennah Konrad, Brian Meisch, Nicolette Athanasiou, Ester Sala, Martí Juanola-Falgarona, Nestor Camenforte, Rebecca Smith-Aguasca, Pau Boher, Sarah Johnson, Matthew L. Faron, Macy G. Wood

**Affiliations:** 1 Department of Pathology, Medical College of Wisconsin 5506, Milwaukee, Wisconsin, USA; 2 STAT-Dx Life, S.L. (a QIAGEN Company), Barcelona, Spain; 3 QIAGEN Manchester 118818 , Manchester, United Kingdom; 4 MDC Associates, Inc, Beverly, Massachusetts, USA; Universitat Munster, Münster, Germany

**Keywords:** *Clostridioides difficile*, *C. difficile*, multiplex testing, diarrhea, stool

## Abstract

Acute gastroenteritis is caused by a variety of pathogens, including bacteria, viruses, and parasites, which may have overlapping clinical signs and symptoms. Community-acquired gastroenteritis is caused by various pathogens, while healthcare-associated gastroenteritis is caused by *Clostridioides difficile*. Current diagnostic approaches utilize *C. difficile* nucleic acid amplification tests (NAATs) alone or in combination with an enzyme immunoassay for *C. difficile* antigen and toxin. This study evaluates the analytical performance of *C. difficile* target detection from 290 clinical specimens using the QIAstat-Dx multiplex gastrointestinal panel (GIP) 2 compared with four commercially available NAATs (Cepheid Xpert *C. difficile*, BD MAX Cdiff, Verigene CDF, and BioFire GI panel) as well as the *C. difficile* immunoassay. The positive percent agreement (PPA) and negative percent agreement (NPA) were calculated for all assays in the study. All molecular assays demonstrated a PPA greater than 85%, and an NPA greater than 96% when compared to the other NAATs. The QIAstat-Dx GIP 2 performance was comparable to the other NAATs, demonstrating 95.0%, 88.5%, 98.1%, and 93.7% PPA and 98.2%, 96.9%, 96.6%, and 99.1% NPA compared to Cepheid Xpert *C. difficile*, BD MAX Cdiff, Verigene CDF, and BioFire GI panel, respectively. Overall, the QIAstat-Dx GIP 2 assay was accurate and comparable to other commercially available methods, and the short assay setup time offers an efficient laboratory stool testing workflow.

**IMPORTANCE:**

*Clostridioides difficile* infection ranges from diarrhea to severe, life-threatening pseudomembranous colitis. Clinical criteria include three or more liquid stools in 24 h, while diagnostic testing includes nucleic acid tests alone or in combination with an enzyme immunoassay for *C. difficile* antigen and toxin. This study evaluates the analytical performance of *C. difficile* detection from clinical specimens using the QIAstat-Dx multiplex gastrointestinal panel compared with four commercial assays (Cepheid Xpert *C. difficile*, BD MAX Cdiff, Verigene CDF assays, and BioFire GI panel) and a *C. difficile* immunoassay. The BioFire GI panel had the highest positive percent agreement (90–100%), followed by the QIAstat-Dx (88–98%), BD MAX (87–98%), and the Xpert assays (86–96%). The QIAstat-Dx demonstrated high positive and negative agreement with all nucleic acid tests. The short assay setup time for the QIAstat-Dx GIP 2 also offers a streamlined testing workflow.

## INTRODUCTION

Acute gastroenteritis (AGE) is characterized by symptoms such as diarrhea, fever, abdominal pain, loss of appetite, nausea, and colitis. The CDC estimates nearly 200 million cases of AGE annually in the United States (1). AGE is caused by a variety of pathogens, including parasites, bacteria, and viruses, often with overlapping clinical manifestations, which makes diagnosis challenging (2). Community-acquired gastroenteritis is caused by various pathogens (1), while healthcare-associated gastroenteritis is due to *Clostridioides difficile* (3).

*C. difficile*, a toxin-producing, anaerobic, gram-positive bacterium, is responsible for approximately 453,000 cases and 29,300 associated deaths, annually (3). The economic burden of *C. difficile* infection (CDI) is substantial, with excess medical costs estimated at $4.8 billion (4). CDI ranges from diarrhea to severe, life-threatening pseudomembranous colitis (5). In healthy adults, the colonic bacterial flora typically confers resistance to *C. difficile* colonization (6). However, disruption of the normal colonic flora, most commonly due to antibiotic exposure, compromises this resistance (7). The primary virulence factor of *C. difficile* is cytotoxin B (8), with the genes encoding toxin A (*tcdA*, an enterotoxin) and toxin B (*tcdB*) located within the pathogenicity locus (PaLoc) (9–11).

Clinical criteria for CDI include three or more liquid stools in 24 h. The gold standard for toxigenic *C. difficile* detection is via toxigenic stool culture or cell cytotoxicity neutralization assays (12, 13). Toxigenic stool cultures and cell cytotoxicity neutralization assays are sensitive but time-consuming and impractical for routine use in most clinical laboratories. Current diagnostic approaches utilize *C. difficile* toxin gene (*tcdA* and/or *tcdB*) nucleic acid amplification tests (NAATs) alone or in combination with an enzyme immunoassay (EIA) for *C. difficile* glutamate dehydrogenase (GDH) antigen and toxin (TcdA and TcdB) (14). *C. difficile* is also a target on commercially available gastrointestinal pathogen panels (GPPs), such as the QIAstat-Dx gastrointestinal panel (GIP) 2 and Luminex xTAG GPP, that simultaneously detect multiple organisms for the evaluation of AGE (15). Although NAATs are highly sensitive and rapid, inappropriate use can result in the overdiagnosis of CDI due to the detection of asymptomatic carriers (16). Consequently, it is important to perform CDI testing only when clinically indicated to inform timely treatment decisions for clinical care.

This study evaluates the analytical performance of *C. difficile* target detection from clinical specimens using a research use only (RUO) version of the QIAstat-Dx GIP 2 compared with four commercially available assays, including the Cepheid Xpert *C. difficile*, BD MAX Cdiff, Verigene CDF assays, and BioFire FilmArray GI Panel.

## MATERIALS AND METHODS

### Clinical specimens

Stool specimens were enrolled at the Medical College of Wisconsin, Milwaukee, WI. Residual stool from subjects of all ages who were submitted to the laboratory for the standard of care (SOC) *C. difficile* testing were enrolled if they met the following criteria: sufficient volume (≥8 mL or ≥6 g), unpreserved and unformed (liquid or soft) stool (Bristol scale score 5–7) in a sterile container, and the ability to be enrolled within 24 h of collection. Formed stools were rejected for *C. difficile* testing. Per institutional policy, specimens were rejected for repeat SOC testing if they were either positive for *C. difficile* within 14 days, or tested negative for *C. difficile* within 7 days, and were excluded from the study.

A waiver of the requirement for informed consent was obtained from the Medical College of Wisconsin institutional review board (IRB) for the use of residual specimens and to collect subject information from their medical records (IRB:PRO00046636). Demographic data were collected at the time of specimen collection, including the results of the clinician-ordered (SOC) *C. difficile* testing (Cepheid Xpert), subject sex, and subject age category. Stools that met the study inclusion criteria were de-identified by an honest broker. Stool consistency was scored using the Bristol scale, then specimens were divided into two containers, one sterile container (unpreserved stool) for testing with the Cepheid Xpert *C. difficile* (SOC test), Verigene CDF, and BD MAX C. diff assays, and one with stool in Meridian Para-Pak C&S Cary-Blair transport medium (preserved stool) for testing with the QIAstat-Dx assay and FilmArray GI Panel. All specimens were stored at 2–8°C upon receipt into the clinical laboratory, and enrolled specimens were tested within 2 days of sample collection. One additional 0.5 mL aliquot was frozen (−70°C) and shipped to STAT-Dx Life (a QIAGEN Company), Barcelona, Spain on dry ice for PCR and bi-directional sequencing analysis.

To supplement the study, 17 previously positive (Xpert), unpreserved, frozen stool specimens were obtained from an independent laboratory and tested by all comparator assays. Demographic data on patient age and gender for this subset of specimens were also obtained. In total, 290 residual clinical stool specimens (273 prospective and 17 retrospective) were evaluated in this study.

### QIAstat-Dx GIP 2 assay

The QIAstat-Dx GIP 2 is a multiplex nucleic acid test designed to detect up to 22 (24 targets including *stx1/2*, *E. coli* O157 serogroup) bacterial, viral, and parasitic pathogens (STAT-Dx Life). During this study, a Research Use Only (RUO) version of the QIAstat-Dx GIP 2 was used to evaluate the *C. difficile* target (detects *C. difficile* toxin A and B gene targets, *tcdA* and *tcdB*, respectively). Testing was conducted according to the manufacturer’s instructions. Briefly, 200 µL of preserved stool in Cary-Blair transport medium was loaded into the QIAstat-Dx *C. difficile* RUO assay cartridge, which was then processed using the modular QIAstat-Dx Analyzer 1.0. The cartridges contain all the necessary reagents for sample preparation, multiplex RT-real-time PCR, and an internal control (IC). A positive signal for the IC indicates a valid assay result, while the lack of a signal on negative results invalidates the assay. The total assay run time is less than 1.5 hs, with an assay setup time of 2 min.

### BioFire FilmArray Gastrointestinal (GI) panel

The BioFire GI panel is a multiplex nucleic acid test designed to detect 22 of the most common pathogens associated with gastroenteritis, including 5 viruses, 4 parasites, and 13 bacteria (BioMerieux, Marcy-l'Étoile, France). The BioFire GI panel detects *C. difficile* gene targets, *tcdA* and *tcdB*. Testing was conducted according to the manufacturer’s instructions. A 200 µL aliquot of stool preserved in Cary-Blair transport medium was mixed with a specialized buffer and loaded into a single-use pouch and run on the BIOFIRE Torch system. The assay setup time requires about 2 min of hands-on time and an approximate run time of 1 h.

### Cepheid Xpert *C. difficile* assay

The Cepheid Xpert *C. difficile* assay (Cepheid, Sunnyvale, CA) is a rapid, automated test for the qualitative detection of toxin-producing *C. difficile* directly from unformed (unpreserved) stool swabs of patients suspected of having CDI. The assay targets and detects *tcdB* and is performed on the Cepheid GeneXpert Dx System (Cepheid). The total assay run time is around 43 min. The assay setup time is approximately 5 min (individual laboratory experience).

### BD MAX Cdiff assay

The BD MAX *C. difficile* assay (BD, Franklin Lakes, NJ) is a real-time polymerase chain reaction (PCR) test designed to detect the *C. difficile tcdB* in unpreserved human liquid or soft stool specimens. The assay amplifies the *tcdB* using PCR and employs fluorogenic target-specific hybridization probes for detecting the amplified DNA. For testing, a disposable 10 µL inoculating loop is dipped into the stool material, and the specimen is dispersed into a BD MAX Cdiff Sample Buffer Tube. The Sample Buffer Tube, BD MAX Cdiff Unitized Reagent Strip, and BD PCR cartridge are loaded onto the BD MAX System, which automates sample preparation, including target lysis, DNA extraction and concentration, reagent rehydration, and target nucleic acid amplification and detection using real-time PCR. The total assay run time is 2 h, with an assay setup time of 1 min, according to the manufacturer’s website.

### Verigene *Clostridium difficile* nucleic acid test (Verigene CDF test)

The Verigene *C. difficile* (Nanosphere, Northbrook, IL) nucleic acid test is a multiplexed, qualitative assay for the rapid detection of three *C. difficile* targets: *tcdA*, *tcdB*, and *tcdC*. This assay will report as *C. difficile* detected if either *tcdA* or *tcdB* is detected; *tcdC* is an optional target for epidemiological purposes to evaluate the presence of PCR ribotype 027 strains. Unformed (unpreserved) stool swabs from patients are processed and diluted according to the test protocol and then applied to the Verigene test cartridge, which is subsequently placed into the Verigene system. The total assay run time is <2 h, with an assay setup time of <5 min according to the manufacturer’s website.

### Bi-directional sequencing of *C. difficile* toxin B gene (*tcdB*)

Sixty-nine clinical specimens positive by any assay (*n* = 71 total positive) were analyzed by *tcdB* PCR, and all PCR-positive specimens underwent bi-directional sequencing for *tcdB*. A segment of the toxin B gene, *tcdB*, was amplified by PCR for bi-directional sequencing. Briefly, *tcdB* was amplified and sequenced using the methods described by Griffiths et al. (17). Nucleic acid extraction of all samples was performed using an automatic purification procedure with the QIAcube System (QIAGEN N.V., Hilden, Germany) and a customized protocol using the reagents of QIAamp Cador Pathogen Mini Kit (QIAGEN, catalog number 54106). One positive control and negative control in triplicate (AVE buffer (166020921; QIAGEN)) were included in every run to confirm amplification and that there was no cross-contamination, respectively. All PCR runs were performed using the QuantStudio 5 Real-Time PCR systems and the QuantStudio Design and Analysis software version 1.4 (Thermo Scientific). All results were analyzed by TapeStation using D1000 ScreenTape (Agilent Technologies, Ref: 5067-5582) with D1000 Reagents (Agilent Technologies, Ref: 5067-5583). All PCR products showing the expected band size of 688 bp in TapeStation were purified by ExoSAP-IT Express PCR Product Cleanup (Thermofisher; Ref: 75001.40 .UL), quantified by Qubit dsDNA high sensitivity assay kits (Thermofisher; Ref: Q32854) and normalized to a concentration of 25–100 ng/µL. Sanger sequencing reactions were assembled with the forward and reverse primers used for amplification at 5 µM using BigDye Terminator v3.1 Cycle Sequencing Kit (Thermofisher; Ref: 4337455) and performed on either 3730 DNA Analyzer or 3730xl DNA Analyzer sequencer (Applied Biosystems). All steps from PCR product cleanup to Sanger sequencing were performed by the Genomics Unit from Scientific and Technological Centers (CCiTUB), Universitat de Barcelona. Sequences were analyzed using Geneious software v.2023.2 (https://www.geneious.com/) and those showing at least 200 bases of an acceptable quality, defined as a minimum of 90% of the total bases with PHRED quality score of 20 or higher and less than 5% of total bases with ambiguous nucleotides, were run on BLAST (https://blast.ncbi.nlm.nih.gov/Blast.cgi) against the non-redundant nucleotide collection (Nr/nt) data base from The National Center for Biotechnology Information (NCBI) database (https://www.ncbi.nlm.nih.gov/) using the standard BLASTn search parameters. The best BLAST hits obtained for the sequences analyzed that had at least 95% query coverage and 95% identity compared to the reference and with a BLAST *e*-value below 1 × 10^−30^ were used to confirm that the sequences corresponded to the *C. difficile tcdB* gene. These sequences were also visually inspected and classified, when possible, into the different *tcdB* subtypes described in Shen et al., based on sequence homology (18).

### C. DIFF QUIK CHEK complete assay

The C. DIFF QUIK CHEK Complete test (Techlab, Blacksburg, VA) is a rapid membrane EIA for the simultaneous detection of *C. difficile* glutamate dehydrogenase antigen and toxins A and B in a single reaction well. Stool specimens were tested as per the manufacturer’s instructions. Seven specimens were not tested for toxin and GDH antigen.

### Data analysis and invalid specimens

For dual target assays, detection of *tcdA* and/or *tcdB* results in a positive *C. difficile* result, whereas assays that target *tcdB* only require detection of *tcdB* for a positive *C. difficile* result (see Table 1 for assay-specific *C. difficile* targets). Testing on each platform was repeated once if a valid result was not obtained. If repeat testing yielded an invalid result a second time, the test was reported as “invalid.” Invalid results included internal control failures, an indeterminate result, a “no call” result, or a system error (e.g., assay platform mechanical failure). Following the manufacturer’s instructions, QIAstat-Dx specimens with an error message to repeat the specimen with 100 µL of the specimen, rather than 200 µL, were performed as indicated. The positive percent agreement (PPA) and negative percent agreement (NPA) were calculated for each assay in comparison with all the other assays in the study. Invalid specimen results were excluded from this analysis.

**TABLE 1:** *C. difficile* assay characteristics

Assay method	Assay type [*n*]^*a*^	*C. difficile* gene target/s	Assay setup time^*b*^	Run time^*b*^	Specimen volume
QIAstat-Dx GIP 2	Multiplex panel [24]	*tcdA*; *tcdB*	2 m	<1.5 h	200 µL
Cepheid Xpert^*c*^	*C. difficile-*specific [1]	*tcdB*	5 m	43 m	Swab
BD MAX Cdiff assay	*C. difficile-*specific [1]	*tcdB*	2 m	2 h	10 µL
Verigene CDF test	*C. difficile-*specific [3]	*tcdA*; *tcdB*; *tcdC^d^*	<5 m	2 h	Swab
BioFire GI panel	Multiplex panel [22]	*tcdA*; *tcdB*	2 m	<1 h	200 µL

^*a*^ “Multiplex” indicates additional bacterial, viral and/or parasite targets; [*n*] number of analytes per assay.

^*b*^ hours (h), minutes (m).

^*c*^ Standard of care (SOC) assay.

^*d*^ Differentiates* tcdA* and *tcdB* targets; *tcdC* is for epidemiological purposes only.

## RESULTS

### Clinical demographics

Prospectively collected, unpreserved, stool specimens (*n* = 273) submitted to the clinical laboratory for the SOC *C. difficile* testing were enrolled in the study. Patient sex, age, and Bristol stool score were recorded for all prospectively enrolled subjects. In addition, demographic data were also obtained (age and sex) from the enrolled subjects for the 17 previous *C. difficile* positive (Xpert) specimens. Just over half of the subjects enrolled in the study were female (54%; *n* = 156/290), with 46% male subjects. The average subject age was 59 (range: 17 to ≥89), with a median age of 62. The Bristol stool score for specimens enrolled in this study ranged from type 5 (soft) to type 7 (entirely liquid). Type 7 stools (*n* = 178) accounted for 65.2% of stool specimens enrolled in the study, followed by type 6 (23.1%; *n* = 63), and type 5 (11.7%; *n* = 32).

### Test performance

Stool specimens enrolled in the study (*n* = 273 prospective; *n* = 17 supplementary positive samples) were evaluated on each of the five NAATs: QIAstat-Dx GIP 2, Cepheid Xpert *C. difficile*, BD MAX Cdiff, Verigene CDF assays, and BioFire FilmArray GI Panel (Table 1). Stool specimens were also tested on the C. DIFF QUIK CHEK Complete *C. difficile* antigen and toxin EIA.

During this study, there were no invalid or failed Cepheid Xpert *C. difficile* tests. Five BioFire specimens (5/290; 1.7%) were invalid due to instrument failure or instrument restart, and six BD MAX specimens (6/290; 2.1%) yielded indeterminate results. Twenty QIAstat-Dx specimens (20/290; 6.9%) failed due to instrument, “sample too concentrated” failures, invalid runs (Internal Control failures with at least one analyte detected and the other analytes reported as “invalid”), or other run failures. Nineteen Verigene specimens failed (19/290; 6.6%) due to high background, internal control failure, indeterminate results, or mechanical or cartridge issues. After a single repeat test for the invalid specimens, both BioFire and BD MAX assays yielded 100% valid results, and only two specimens remained invalid (0.7% overall invalid rate) on the QIAstat-Dx and Verigene.

### Agreement between NAATs

In this study, there were 71 specimens that were positive on at least one NAAT. Forty-nine specimens (49/290; 16.9%) were positive on all five NAATs and seven additional specimens were positive by four of the five NAATs. The BioFire GI panel had the greatest number of positive results (*n* = 63), followed by the QIAstat-Dx GIP 2 (*n* = 61) and BD MAX Cdiff (*n* = 61), then Cepheid Xpert *C. difficile* (*n* = 60), Verigene CDF (*n* = 53), and the *C. difficile* antigen (*n* = 48) and toxin (*n* = 19) assay (Table 2).

**TABLE 2:** Positive percent agreement^*c*^^,^^*d*^

*n* = 290		Comparator method						
		Cepheid Xpert	BD MAX Cdiff	Verigene CDF^*a*^	QIAstat-Dx GIP 2^*a*^	BioFire GI panel	*C. difficile* Ag^*b*^	*C. difficile* toxin^*b*^
Positive (*n*)		60	61	53	61	63	48	19
Test method	Cepheid Xpert		53/61	51/53	57/61	59/63	44/48	19/19
			**86.9%**	**96.2%**	**93.4%**	**93.7%**	**91.7%**	**100.0%**
	BD MAX Cdiff	53/60		52/53	54/61	55/63	40/48	19/19
		**88.3%**		**98.1%**	**88.5%**	**87.3%**	**83.3%**	**100.0%**
	Verigene CDF^*a*^	51/60	52/61		52/60	53/62	39/47	19/19
		**85.0%**	**85.2%**		**86.7%**	**85.5%**	**83.0%**	**100.0%**
	QIAstat-Dx GIP 2^*a*^	57/60	54/61	52/53		59/63	44/48	19/19
		**95.0%**	**88.5%**	**98.1%**		**93.7%**	**91.7%**	**100.0%**
	BioFire GI panel	59/60	55/61	53/53	59/61		44/48	19/19
		**98.3%**	**90.2%**	**100.0%**	**96.7%**		**91.7%**	**100.0%**
	*C. difficile* Ag^*b*^	44/58	40/61	39/51	44/59	44/61		18/19
		**75.9%**	**65.6%**	**76.5%**	**74.6%**	**72.1%**		**94.7%**
	*C. difficile* Toxin^*b*^	19/58	19/61	19/51	19/59	19/61	18/48	
		**32.8%**	**31.1%**	**37.3%**	**32.2%**	**31.1%**	**37.5%**	

^*a*^ Two specimens were “no call” or failed.

^*b*^ Seven specimens were not tested.

^*c*^ Percent agreement (bold format) calculated from raw data.

^*d*^ Gray boxes indicate comparator and test method are the same method.

The PPA and NPA were calculated for each assay against the other as the comparator method (Tables 2 and 3). The tables include both the raw data and the percent agreement for each paired comparison. If the result for one test in the paired comparison was invalid, both test results were excluded from the analysis (i.e., the denominator changed based on the number of valid test results). The BioFire GI panel had the highest overall PPA (90–100% PPA), followed by the QIAstat-Dx GIP 2 (88–98% PPA), the BD MAX (PPA 87–98%), and the Xpert *C. difficile* assay (86–96% PPA), when compared to the other NAATs (Table 2). The Verigene CDF PPA ranged from 85% to 86%, compared to the other NAATs (Table 2). The NPA was greater than 96% for all NAATs (Table 3).

**TABLE 3:** Negative percent agreement^*c*^^,^^*d*^

*n* = 290		Comparator method						
		Cepheid Xpert	BD MAX Cdiff	Verigene CDF^*a*^	QIAstat-Dx GIP 2^*a*^	BioFire GI panel	*C. difficile* Ag^*b*^	*C. difficile* toxin^*b*^
Negative (*n*)		230	229	235	227	227	235	264
Test method	Cepheid Xpert		222/229	226/235	224/227	226/227	221/235	225/264
			**96.9%**	**96.2%**	**98.7%**	**99.6%**	**94.0%**	**85.2%**
	BD MAX Cdiff	227/230		231/235	225/227	226/227	221/235	229/264
		**98.7%**		**98.3%**	**99.1%**	**99.6%**	**94.0%**	**86.7%**
	Verigene CDF^*a*^	226/228	226/227		225/226	226/226	222/234	230/262
		**99.1%**	**99.6%**		**99.6%**	**100.0%**	**94.9%**	**87.8%**
	QIAstat-Dx GIP 2^*a*^	224/228	220/227	225/233		223/225	218/233	222/262
		**98.2%**	**96.9%**	**96.6%**		**99.1%**	**93.6%**	**84.7%**
	BioFire GI panel	226/230	221/229	226/235	223/227		218/235	222/264
		**98.3%**	**96.5%**	**96.2%**	**98.2%**		**92.8%**	**84.0%**
	*C. difficile* Ag^*b*^	221/225	216/224	222/230	218/222	218/222		234/264
		**98.2%**	**96.4%**	**96.5%**	**98.2%**	**98.2%**		**88.6%**
	*C. difficile* Toxin^*b*^	225/225	224/224	230/230	222/222	222/222	234/235	
		**100.0%**	**100.0%**	**100.0%**	**100.0%**	**100.0%**	**99.6%**	

^*a*^ Two specimens were “no call” or failed.

^*b*^ Seven specimens were not tested.

^*c*^ Percent agreement (bold format) calculated from raw data.

^*d*^ Gray boxes indicate comparator and test method are the same method.

The QIAstat-Dx GIP 2 assay (preserved stool) demonstrated 95.0%, 88.5%, 98.1% PPA (Table 2) and 98.2%, 96.9%, 96.6% NPA (Table 3) compared to unpreserved stool specimens tested by the Cepheid Xpert *C. difficile*, BD MAX Cdiff, and the Verigene CDF assay, respectively. Using preserved specimens, the QIAstat-Dx GIP 2 demonstrated 93.7% PPA and 99.1% NPA compared to the BioFire GI panel (Tables 2 and 3).

### *C. difficile* toxin and antigen (Ag) evaluation

The number of *C. difficile* toxin and antigen (GDH)-positive specimens was lower compared to *C. difficile* target detection by NAATs. Of the specimens tested, 19 were positive for *C. difficile* toxin and 48 were positive for GDH. All 19 *C*. *difficile* toxin-positive specimens were also positive for the *C. difficile* target each of the five NAATs evaluated in the study. The PPA for GDH-positive specimens compared to NAAT detection of *C. difficile* ranged from 65% to 76% (Table 2), while the NPA was 96–98% compared to the NAATs (Table 3). Three stool specimens in the study were GDH-positive, toxin-negative, and negative for *C. difficile* toxin genes by all five NAATs.

### Bi-directional sequencing and *tcdB* subtyping analysis

In this study, 71 specimens were positive by at least one NAAT. Sixty-nine clinical specimens reporting at least one NAAT *C. difficile* positive result were analyzed by *tcdB* PCR followed by bi-directional sequencing to subtype the *C. difficile* toxin B gene. Two specimens were not tested by PCR and sequencing; one was positive by four of the five NAATs (Xpert, QIAstat-Dx, BioFire, and BDMax but negative on the Verigene) and the other was positive only on the QIAstat-Dx. Of the 69 specimens tested by PCR, 11 were negative (did not amplify a product by PCR), and 1 specimen did not provide sequence data. Six of the *tcdB* PCR negative specimens were only *C. difficile* positive by the BD MAX assay; two specimens were positive by two NAATs (one BioFire and QIAstat; one Xpert and QIAstat positive), one was positive by three NAATs (Xpert, QIAstat, and BioFire), and two were *C. difficile* positive by all five NAATs.

The remaining 57 specimens were *tcdB* positive by PCR and sequencing analysis (Table 4). Compared to bi-directional sequencing confirmed *tcdB*, the PPA was highest for the BioFire (100%), followed by the Cepheid Xpert (94.7%), QIAstat-Dx (93.0%), BD MAX (89.5%), and Verigene (87.7%). The predominant subtype was *tcdB1a* (77%; *n* = 44/57), followed by *tcdB2* (15.8%; 9/57), and *tcdB4* (3.5%; 2/57). The subtype could not be determined in 3.5% (2/57) of specimens tested.

**TABLE 4:** Positive percent agreement with *tcdB* PCR and bi-directional sequencing analysis^*a*^

Assay	Positive (*n* = 57)	Percent (%)
BioFire GI panel	57	100.0%
Cepheid Xpert	54	94.7%
QIAstat-Dx GIP 2	53	93.0%
BD MAX Cdiff	51	89.5%
Verigene CDF	50	87.7%
*C. difficile* Ag	42	73.7%
*C. difficile* toxin	19	33.3%

^*a*^ Sixty-nine specimens were tested by PCR; 11 specimens were PCR negative, and 1 PCR-positive specimen did not yield sequencing data.

## DISCUSSION

NAATs have been available for more than a decade for the rapid detection of *C. difficile* toxin genes directly from clinical stool specimens and have largely replaced cytotoxicity assays or toxigenic cultures (19, 20). This study evaluated the analytical performance of five NAATs for the detection of *C. difficile*, including two multiplex GI panels. The performance of the QIAstat-Dx GIP 2 assay was comparable to other commercially available *C. difficile* NAATs (Tables 2 to 4). Of the specimens that yielded sequencing data, the predominant *tcdB* subtype was *tcdB1a* (77%; *n* = 44), followed by *tcdB2* (15.8%), and *tcdB4* (3.5%), which is consistent with published reports that *tcdB1* (69.9%) and *tcdB2* (26.9%) are more common in North America, while *tcdB1* (66.3%) or *tcdB3* (29.5%) are reported in East Asia (18).

The BioFire detected the highest number of *C. difficile* positive specimens (*n* = 63), whereas the Verigene detected the least in this study (*n* = 53). Variable assay target/s (i.e., t*cdB* vs *tcdA/B*), reported limit of detection (LoD), and/or specimen volume likely impact assay-specific detection rates. For the NAATs that detect multiple *C. difficile* targets (e.g., QIAstat, Verigene, and BioFire), the detection of *tcdA* or *tcdB* will result in a “positive *C. difficile*” interpretation, so differences could be related to the absence of *tcdB* in a specimen (see pooled data analysis below). The reported LoD for each NAAT varied based on the *C. difficile* strain tested by the manufacturer (21). The Verigene LoD ranged based on the strain tested (63–1,250 colony forming units [CFU]/mL) with the final LoD defined as 1,250 CFU/mL, compared to QIAstat-Dx, Xpert, and BD MAX, which reported a lower LoD (i.e., 41–832 CFU/mL) (21). The Verigene utilizes a stool swab, which may also contribute to the higher LoD and could explain the lowest number of positive specimens detected in this study. Of note, six specimens in this study were positive only on the BD MAX assay (see discussion below); if those results were excluded as potential false positives, the BD MAX data (*n* = 55) would more closely align with the overall number of positive Verigene results (*n* = 53).

Compared to specimens that were confirmed by *tcdB* PCR and sequencing (*n* = 57), the BioFire, Xpert, and QIAstat-Dx demonstrated high agreement ≥93% (Table 4). Of the 69 specimens tested, 1 specimen with a light PCR band did not yield sequence data, and 11 did not amplify a PCR product. The sample with a light PCR band was positive on all five NAATs in the study but yielded a high cycle threshold (Ct) value (Ct = 31.9 and 30.6 on the Xpert and QIAstat, respectively), which suggests that the negative sequencing results are likely more reflective of a low DNA burden in the sample that negatively impacted sequencing. Six of the 11 specimens were positive only on the BD MAX; each specimen was negative for GDH, toxin, and *tcdB* PCR. Five of the six specimens were re-run using a fresh aliquot and yielded *C. difficile* negative results, which may support initial false-positive results. The remaining five *tcdB* PCR-negative specimens were positive on multiple NAATs. Two specimens were positive by all five NAATs and had high Ct values (specimen one Ct = 31.1 and Ct = 25.6; specimen two Ct = 32.8 and Ct = 27.9 on the Xpert and QIAstat assays, respectively). Three specimens were *C. difficile* positive on two or more of the NAATs. One specimen was positive on the Xpert (Ct = 37.0), QIAstat (Ct = 31.9), and BioFire assays, while the second specimen was positive on both the Xpert (Ct = 28.2) and QIAstat (Ct = 32.4), and a third specimen was positive on the QIAstat (Ct = 31.5) and BioFire assays. Low *C. difficile* DNA abundance may also have negatively impacted PCR and sequencing efforts for these specimens. Overall, false-positive results appear less likely for this subset of five specimens due to the repeated *C. difficile* detections by multiple different NAATs in the study.

Consistent with published reports, lower agreement was observed between the *C. difficile* GDH antigen assay and NAAT detection of *C. difficile* (65–76% PPA) (22), but a high NPA (>96%). This is not unexpected due to the difference in analytical sensitivity of an immunoassay compared to a NAAT (23, 24). Three specimens in the study were GDH antigen positive, but *C. difficile* toxin negative and *C. difficile* negative by all NAATs, which suggests the absence of *C. difficile* toxin genes. Between 3% and 30% of *C. difficile* isolates are reported to be non-toxigenic (25, 26), but this varies considerably based on study location, patient population, symptomatic versus asymptomatic patients, and the type of laboratory testing performed (27).

### Pooled assay agreement

The gene targets for *C. difficile* detection differ between the NAATs evaluated in this study. The Xpert and BD MAX target the *tcdB* gene (single target)*,* while the remaining NAATs (QIAstat-Dx, BioFire, Verigene) detect both *tcdA* and *tcdB* genes (dual target). To calculate pooled *C. difficile* positive results by single or dual target assay, any assay with at least one positive result was considered positive for *C. difficile*. Pooled results for the assays that target only *tcdB* yielded 68 *C*. *difficile* positive specimens, whereas the dual target, *tcdA/B* assays, detected 66 *C*. *difficile* positive specimens. Five specimens were positive by *tcdB* only assays; all five were positive only on the BD MAX. As discussed above, these five were likely false positives upon initial testing.

Three specimens in the study were positive only by the pooled, dual target (*tcdA/B*) assays (one QIAstat-Dx and BioFire positive, one BioFire only positive, and one QIAstat-Dx only positive specimen). These discrepancies could be due to the presence of *C. difficile* strains that lack *tcdB* but contain *tcdA* (e.g., *tcdA+/tcdB*−) or due to *tcdB* mutations in the region/s targeted by the assays. Using the QuikChek and *tcdB* PCR and bi-directional sequencing data, the positive QIAstat/BioFire specimen was GDH positive, toxin negative, and the *tcdB* PCR did not result in product amplification, which suggests the absence of *tcdB* in this specimen. The other two single assay positives were negative for both GDH and toxin. However, the positive BioFire specimen harbored *tcdB1a* by PCR and bi-directional sequencing, which indicates that it is a false-negative on all other NAATs. The remaining specimen was positive only on the QIAstat-Dx but was not further evaluated in this study.

The inclusion of *tcdA* as an additional *C. difficile* target may reduce false-negative *C. difficile* results in *C. difficile* strains with *tcdB* mutations. However, previous publications have demonstrated that Toxin B is an essential virulence factor for CDI (11, 28), and *C. difficile* strains that lack *tcdB* are less virulent than strains with *tcdB alone* or *tcdA* and *tcdB* (29). Consequently, NAATs with dual *tcdA/B* targets could potentially result in overtreatment of *C. difficile tcdA^+^/tcdB^−^* strains.

### Processing and TAT

The QIAstat-Dx assay requires 2 min of hands-on time for assay setup and the assay run time is less than 1.5 h. The setup time for QIAstat-Dx is comparable to the other assays in this study, which require approximately 1–5 min for setup and the assay run time ranges from 43 min to 2 h.

### Assay invalid rates/indeterminate results

The initial failure rates for the QIAstat-Dx, Verigene CDF, BD MAX, BioFire, and Cepheid tests were 6.9%, 6.6%, 2.1%, 1.7%, and 0%, respectively, which is consistent with published reports (19, 20). After repeat testing, only two (0.7%; 2/290) specimens on the Verigene and the QIAstat-Dx remained invalid. No correlation between invalid results and specimen characteristics (e.g., mucoid or excessively bloody) was observed.

### Study limitations

Stool specimens were prospectively enrolled from clinical specimens submitted for *C. difficile* SOC testing at a single academic hospital. Specimens were accepted for testing if stool conformed to the shape of the container and was ≥5 on the Bristol scale. *C. difficile* test orders are not restricted at the performing institution aside from the rejection of formed stools. The laboratory information system blocks repeat testing within 7 days of a negative result or 14 days of a positive result. Finally, cytotoxic testing, the gold standard for CDI, was not performed during this study (13).

Importantly, diagnosis of CDI requires clinical symptoms consistent with CDI and cannot rely solely on laboratory testing (16). A chart review was not performed to evaluate for clinical symptoms consistent with CDI. Several published studies have evaluated the use of Ct values to distinguish *C. difficile* colonization versus infection (reviewed in reference 30), but other studies demonstrated overlapping Ct values between both groups when using PCR compared to cell cytotoxicity neutralization assays (31). Caution is warranted for the use and interpretation of Ct values as values will likely vary based on the assay (variables include stool input volume, assay extraction and amplification methodology, *C. difficile* target and copy number, etc.). However, intra-assay variability may be less of a concern; using the QIAstat-Dx GIP 2 assay, reproducibility and linearity for quantification of *C. difficile* burden were recently demonstrated (32). In the present study, only the Xpert and QIAstat-Dx NAATs yielded Ct values, and correlation between the two assays was observed (*R*^2^ = 0.7992; data not shown). To mitigate overdiagnosis, some laboratories may choose to selectively report the *C. difficile* target from multiplex panels (33), while other studies report this strategy may lead to missed diagnoses and associated morbidity and mortality (34), further highlighting the complexity of diagnostic testing for CDI.

Overall, the QIAstat-Dx GIP 2 assay is comparable to the commercially available nucleic acid amplification assays for the detection of *C. difficile*, and the rapid assay setup time offers an efficient laboratory testing workflow.
